# An efficiency framework for valence processing systems inspired by soft cross-wiring

**DOI:** 10.1016/j.cobeha.2016.08.002

**Published:** 2016-10

**Authors:** P Read Montague, Kenneth T Kishida, Rosalyn J Moran, Terry M Lohrenz

**Affiliations:** 1 Virginia Tech Carilion Research Institute & Dept Physics, Virginia Tech, USA; 2 The Wellcome Trust Centre for Neuroimaging, University College London, WC1N 3BG, UK; 3 Department of Engineering Mathematics, University of Bristol, Bristol BS8 1UB, UK

## Abstract

Recent experiments suggest that subsecond dopamine delivery to human striatum encodes a combination of reward prediction errors and counterfactual errors thus composing the actual with the possible into one neurochemical signal. Here, we present a model where the counterfactual part of these striatal dopamine fluctuations originates in another valuation system that shadows the dopamine system by acting as its near-antipode in terms of spike-rate encoding yet co-releases dopamine alongside its own native neurotransmitter. We show that such a hypothesis engenders important representational consequences where valence processing appears subject to the efficient encoding considerations common to the visual and auditory systems. This new perspective opens up important computational consequences for understanding how value-predicting information should integrate with sensory processing streams.

## Highlights

• Serotonin has been proposed as an opponent to dopamine.
• This review explores positive and negative value pathways for structuring this opponency.
• The positive and negative pathways co-mingle through transmitter cross-loading.
• Cross-loading is proposed as a way to tile ‘valence space.’

**Current Opinion in Behavioral Sciences** 2016, **11**:121–129

This review comes from a themed issue on **Computational modeling**

Edited by **Peter Dayan** and **Daniel Durstewitz**

For a complete overview see the Issue and the Editorial

Available online 8th September 2016

**http://dx.doi.org/10.1016/j.cobeha.2016.08.002**

2352-1546/© 2016 The Authors. Published by Elsevier Ltd. This is an open access article under the CC BY license (http://creativecommons.org/licenses/by/4.0/).

## Counterfactual signaling encoded by striatal dopamine fluctuations

**Figure 1:**
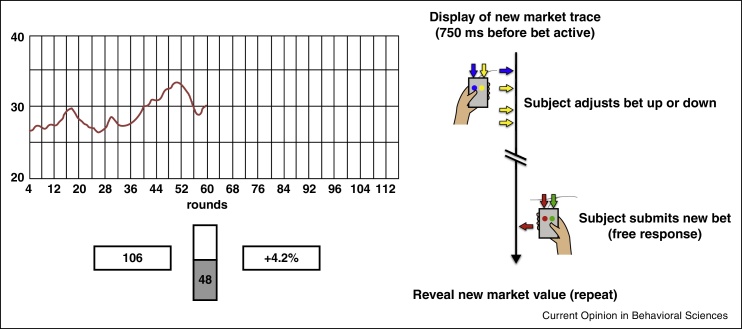
Sequential betting game against a market. Subjects bet between 0% and 100% of their total on each trial, a ‘price’ variable changes (goes up or down), and the subject gains or loses that fractional amount of their current total. There are no time limits between choices [1^•^].

Recent work in human striatum has provided electrochemical evidence that subsecond dopamine fluctuations carry information related to two distinct kinds of error signals — Firstly, reward prediction error (as anticipated by a large literatures in rodents and primates) and finally, counterfactual error encoding [1^•^]. The authors note that the ‘compositional encoding of ‘actual’ and ‘possible’ is consistent with how one should ‘feel’ and may be one example of how the human brain translates computations over experience to embodied states of subjective feeling’ [1^•^]. In contrast with this lofty possibility, we present a computational perspective on the findings that exploits the hypothesis that the counterfactual signals carried by dopamine arise in a paired system that nearly anti-correlates with dopaminergic encoding of prediction errors in reward but is ideally suited to predict future aversive stimuli. The motivating finding is shown in Figure 1.

The idea proposed by Kishida and colleagues was that some other source/sink for dopamine existed that could encode information about foregone gains and losses (encoded in the game by bet level [1^•^]). In the game, subjects saw a stationary price trace, placed a bet expressed as a fraction of their total holdings between 0% and 100%, the price fluctuated to its next value, and losses or gains occurred. Subsecond dopamine measurements in the human striatum encoded signed fluctuations around a running estimate of the mean outcome, but also showed a dependence on the bet level, which suggested that dopamine was encoding a combination of reward prediction errors in outcome (which scales positively with the price fluctuation) and a separate part that scaled negatively with the price fluctuation (which they termed the counterfactual error). There are many counterfactual errors one can define in this simple game, but Kishida and colleagues specifically meant the foregone gains or losses compared to how well or poorly things might have gone in the extreme (bets all in or all out, see [1^•^]).

Several possibilities ensue from these observations. First, it is possible that midbrain dopamine neurons, in the context of this simple cognitive challenge, have prediction error and counterfactual error computations available and encoded appropriately as changes in spike rate. The reason this has not been observed before is that prior work never really challenged an animal in the same way (with parametrically connected foregone gains and losses; see Figure 1) while recording either dopaminergic spikes or dopamine fluctuations at target projection sites. A second possibility, a version of the first, is the existence of another population of dopaminergic neurons (not previously described) that modulate their activity oppositely to dopaminergic neurons and effectively add/subtract dopamine from baseline extracellular levels as a near-opponent to the prediction error encoding long described for these neurons. A third possibility is that some neuronal population that nearly anti-correlates with the dopaminergic modulation during reward-based tasks releases dopamine because it is capable of loading dopamine into its terminals rather than manufacturing it itself, and does so in the same regions of the dorsal striatum (the primary recording site of Kishida and colleagues). This latter possibility falls into the opponent process hypothesis (see [2••, 3]).

To summarize the possibilities: (1) midbrain dopamine neurons known for generating reward prediction error signals also generate spike modulations consistent with prediction error and counterfactual error signaling, OR (2) there is another class of midbrain dopamine neuron dedicated to the counterfactual term, OR (3) there is an opponent to the dopaminergic reward prediction error signal that releases dopamine or controls the release of dopamine in striatal regions. This list is not biologically exhaustive. In this opinion piece, we restrict our focus to the possibility that the shadow system in possibility 3 are serotonergic neurons from the nucleus raphe and we lean on the fact that they can load dopamine into their terminals [4^••^].

## Cross-loading between serotonin and dopamine: inspiring the *P* and *N* model

There is solid neurobiological evidence that dopamine and serotonin are capable of cross-loading into one another's terminals [4••, 5•, 6••]. For example, Zhou *et al.* [6^••^] have provided compelling evidence that, under a multi-week regimen of selective serotonin reuptake inhibitors (SSRI), serotonin loads into dopaminergic terminals through dopamine transporters. Whether this displaces the dopamine carrying capacity of these terminals is not known quantitatively but one class of behavioral side effect of selective serotonin reuptake inhibitors (SSRIs) resembles Parkinsonian symptoms; an observation consistent with a diminishment in dopaminergic transmission. In a another recent report, Gantz *et al.* [5^•^] showed that under l-DOPA treatment, serotonin terminals originating from neurons in the dorsal raphe nucleus contributed directly to dopaminergic transmission. This cross-loading has important downstream consequences including the fact that there are two dynamic sources of dopamine fluctuations — dopaminergic terminals and serotonergic terminals. In the event that the parent dopamine and serotonin neurons encode different operations, these operations would be combined due to cross-loading. This is exactly the possibility that we offered above to explain the human dopamine data recorded in humans during the simple betting game (Figure 1) adding the hypothesis that the counterfactual signal encoded in dopamine is likely being carried by modulation of serotonergic neurons but translated into both dopamine and serotonin co-release.

Below we build on this cross-wiring hypothesis to suggest that the reward prediction system thought to be represented in part by mesostriatal dopaminergic projections is mirrored by an aversive prediction system carried to the same target neural structure by serotoninergic fibers. Furthermore, these systems may mix their computations through neurotransmitter cross-loading, here termed soft cross-wiring to emphasize the computational composition idea rather than just a physiological eventuality. These biophysical possibilities are consistent with the data shown above but not necessitated by them; however, we show that soft-cross wiring also suggests a different way to conceive of valence processing in terms of efficient encoding hypotheses more typical of visual and auditory analyses [7•, 8, 9, 10••, 11•, 12, 13].

To summarize briefly: Humans with Parkinson's disease exhibit subsecond striatal dopamine fluctuations that encode a combination of a reward prediction error signal and a counterfactual error signal with the latter signal type consistent with a near antipode of the reward prediction error (in the restricted case of the simple game used here) [1^•^]. Here, such an antipode of a reward prediction error signal would be an aversive prediction error signal, which for example fluctuates above and below baseline in a fashion nearly opposite to the reward prediction error signal. The simplest way to account for this oppositely directed prediction error is to suppose that this other system is learning to predict future aversive stimuli in a manner analogous to reward prediction accounts typical of dopamine systems [14]. If so, then the soft cross-wiring that we sketched above has new and very interesting consequences for valence processing in general.

## Separating *P* and *N* error signaling from neurotransmitter semantics

We pursue these ideas by assuming that there are two neuronal systems, *P* and *N* (positive and negative), capable of learning and emitting prediction errors in future rewards and aversions respectively and suggest that the neurotransmitter couplings between these systems can be seen as one way to transform from separate *P* and *N* systems, let us call that the {*P*,*N*} bases, to a different basis {(*P* + *N*), (*P* − *N*)}, which act respectively as a salience channel (*P* + *N*) and a value contrast (*P* − *N*) channel (see Figure 3). This means that salience processing and value contrast processing would be handled by the combination of dopamine and serotonin and not just one system alone; a fact that may also help to explain the odd relationship between dopamine, salience and reward prediction error signaling generally.

In this section, we first review briefly current reinforcement learning (RL) models [15] of how modulations in spike activity in dopaminergic neurons report on prediction errors in future reward [16, 17, 18] and we build a similar but nearly opposite case for a system that would shadow the dopaminergic system in terms of predicting future aversive stimuli [2^••^]. One new step is to assume that all states can be independently and concurrently assigned positive (reward predicting) and negative (aversive predicting) value.

To learn from experience a mobile organism must possess adaptive mechanisms for valuing the world in the face of changing contingencies; an almost self-evident rendering of what it means to adapt to and learn from a variable world. One general approach to learning about rewarding and aversive events is called reinforcement learning (RL), which focuses on how an agent responds to, stores, and plans actions around the rewards and aversives it encounters or could have encountered [15]. A typical reinforcement learning (RL) account of reward learning in animal brains begins with a simple hypothesis about how an organism should value its future states, and moves on to suggest how, given that model of valuation, the system should update the valuation of its states based on experience (for overviews see [16, 17, 18, 19, 20]). This paper avoids a detailed discussion of how such systems organize the mapping from valuations to actions in order to emphasize the conditions under which our proposal — soft cross wiring — engenders downstream computational consequences.

In reinforcement learning, the main valuation hypothesis is that a learning agent should assign a value *V*^*P*^ to its current state *S*_*t*_ according to the discounted rewards expected from that state into the distant future [15]. Here the superscript *P* indicates positive valence. This simple hypothesis embeds the Markovian or history-independent assumption — how a state is acquired is not relevant to its valuation, only the future that it portends influences its value:

(1) $$V^{P}(S_{t})=E[r_{t}+\gamma r_{t+1}+\gamma^{2}r_{t+2}+\cdots ]$$

*E* is the expected value operator, *γ* is a discount factor set somewhere between 0 and 1 that devalues rewards expected to the future of the current state, and t is discretized time. According to the same idea, the valuation of the next state *S*_*t*+1_ follows similarly:

(2) $$V^{P}(S_{t+1})=E[r_{t+1}+\gamma r_{t+2}+\gamma^{2}r_{t+3}+\cdots ]$$

From these expressions, one arrives at a form of the well-known Bellman equation 4 that relates the value of the state at one moment to the value of the state in the next moment (allowing that we are not specifying any properties of this state transition in this paper):

(3) $$E[r_{t}]+\gamma V^{P}(S_{t+1})=V^{P}(S_{t})$$

If a learning agent (like a rat) was using a similar scheme to value its states then a natural ‘error signal’ would be the difference between the right and left hand sides of Equation 3:

(4) $$\delta_{t}^{P}=E[r_{t}]+\gamma V^{P}(S_{t+1})-V^{P}(S_{t})$$

This kind of error signal can be used simply and directly to update parameters used to estimate the value function:

(5) $$V^{P}(S_{t})\leftarrow V^{P}(S_{t})+\alpha \cdot \delta_{t}^{P}$$

There is now substantial evidence that a subset of mammalian midbrain dopamine neurons encode $\delta_{t}^{P}$ into *perturbations in their spike rate* [16, 17, 18, 19, 20]. Hence, dopamine neurons communicate a spike-rate-change-encoded prediction error $\delta_{t}^{P}$ to their terminals and the *neurotransmitter in those terminals* converts $\delta_{t}^{P}$ to a diffusive signal that communicates through the tissue to appropriately selective downstream effectors (e.g. dopamine receptors). Few if any models have explored the reason or limitations of this dissipative step for the particular case of a reward prediction error $\delta_{t}^{P}$.

An exactly analogous argument could be made for the learning of future aversive stimuli and the way a state should be valued in terms of predicting this discounted aversive future. The hypothesis here is that the potential negative value *V*^*N*^ associated with a state *S*_*t*_ is the expected value of exponentially discounted aversives expected from *S*_*t*_ forward into the distant future:

(6) $$V^{N}(S_{t})=E[a_{t}+\gamma a_{t+1}+\gamma^{2}a_{t+2}+\cdots ]$$

which leads to the same Bellman equation as above (but framed on future aversive stimuli) and yields its own error signal $\delta_{t}^{N}$:

(7) $$\delta_{t}^{N}=E[a_{t}]+\gamma V^{N}(S_{t+1})-V^{N}(S_{t})$$

Which can be used to update the value function over aversives:

(8) $$V^{N}(S_{t})\leftarrow V^{N}(S_{t})+\beta \cdot \delta_{t}^{N}$$

The basic idea for the two systems is that each updates its predictions of future rewards and aversives separately but these predictions combine to produce a composite error signal encoded as signed pertubations in baseline spike rates, $\delta_{t}^{P}$ along the *P* pathway and $\delta_{t}^{N}$ along the *N* pathway, which would translate into signed fluctuations in dopamine and serotonin release. The extracellular space ‘adds up’ the ensuing changes in these transmitters to encode $\delta_{t}^{P}+\delta_{t}^{N}$. Similarly, receptors sensitive to either or both transmitters or that through intracellular signaling converged on common targets could likewise compose these signals in flexible ways. This composite error signal is thus well placed to update an overall value function *V*^*P*^(*S*_*t*_) − *V*^*N*^(*S*_*t*_). This conceptual framing of the valuation and prediction problem (without committing to any specific representation) closely resembles Daw *et al.* [2^••^] except that it possesses two separate value functions and thereby entails two signed prediction error signals. The explicit consideration of the prediction errors as diffusible signals within a common space allows them to act alone or together in a manner dependent only on the response elements present. A new possibility occurs when one considers what happens when one neurotransmitter, say dopamine, carries information related to both prediction errors $\delta_{t}^{P}$ and $\delta_{t}^{N}$. Conversely, each prediction error is encoded as a mixture of serotonin and dopamine.

One important assumption in this account is that neurotransmitter fluctuations (e.g. dopamine, serotonin fluctuations) are already *understood* by downstream receptor systems as updating respectively future predictions about positively valenced and negatively valenced stimuli. So one key conceptual step is to separate the error encoding by the parent neurons (expressed as perturbations in spike rate) from the neurotransmitter semantics (as interpreted by downstream effector mechanisms).

## The ***P*** ± ***N*** basis

We have presented a caricaturized view of valence processing by considering the dopamine system as the positive valence pathway *P* and separately imagining that the serotonin system is the negative valence pathway *N*. We have sketched how *P* and *N* could direct reward and aversive prediction learning in fashion aligned with reinforcement learning models generally, and argued for midbrain dopamine and serotonin systems as a possible substrates. This style of model has been used fruitfully to understand a wide range of behavioral data and has informed possibilities for mapping these models to supporting biological substrates [15, 21, 22, 23, 24]. However, the simple opponency claim for the two systems has serious difficulties. The most glaring is that it appeals to the dopamine and serotonin systems as being near-antipodes to one another and thus apparently redundant. There are many contexts where a resource-constrained system should show redundancy as an inefficiency, and this perspective has been explored for decades in sensory systems[9, 10••, 11•, 12, 13]. We consider this apparent value system redundancy from a different perspective and motivate why such systems might want to share neurotransmitter as the data suggest they do.

**Figure 2:**
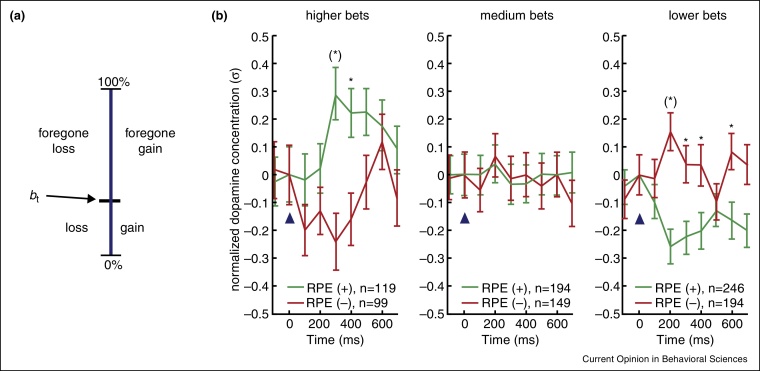
Dopamine fluctuations in the human striatum encode the difference of reward prediction errors and counterfactual errors during a simple betting game. Prediction errors are computed as fluctuations in outcome around a running estimate of the mean outcome (blue triangle indicates time outcome is revealed). Counterfactual errors for this game were defined as the best/worst outcome minus the actual outcome or 1 · *r*_*t*_ − *b*_*t*_*r*_*t*_ = *r*_*t*_(1 − *b*_*t*_) where *r*_*t*_ was the fractional change in price Δ*p*_*t*_/*p*_*t*_ at trial *t*. The hypothesis is that dopamine transients encode a difference between reward prediction errors and this style of counterfactual error. Notice that on gains, *r*_*t*_ is positive and so the counterfactual mechanism would have to subtract dopamine from the extracellular space. On losses, *r*_*t*_ is negative and so the counterfactual mechanism would have to add dopamine to the extracellular space. The dependence of dopamine changes on the bet *b*_*t*_ is captured (qualitatively) by the term. This model (subtracting the counterfactual term suggested above) is equivalent to assuming a separate signal that scales with −*r*_*t*_ and can add/remove dopamine from the extracellular space relative to ongoing baseline levels. The reward prediction error pathway would scale with +*r*_*t*_ and likewise be capable of increasing and decreasing dopamine relative to baseline. Red traces are for outcomes where prediction errors were negative and green traces are for outcomes where prediction errors were positive (error bars are ±SEM; see [1^•^] for statistical details). At high bets, the counterfactual term drops to 0, but grows as bets decrease, an effect that would add/subtract dopamine depending on the sign of the RPE.

As suggest in Figure 2, the *P* and *N* pathways represent separate positive and negative valence prediction capacity. Here we show a depiction of the bi-directionally coupled *P* and *N* systems where perturbations in the dynamics of the two neurotransmitters (*δD*(*t*), *δS*(*t*)) are controlled by both the spike-encoded reward prediction errors $\delta_{t}^{P}$ and the spike-encoded aversive prediction errors $\delta_{t}^{N}$:

(9) $$\left(\begin{array}{c} \delta D\left(t\right)\\ \delta S\left(t\right) \end{array}\right)=\left(\begin{array}{cc} \alpha & 1-\beta \\ 1-\alpha & \beta \end{array}\right)\left(\begin{array}{c} \delta_{t}^{P}\\ \delta_{t}^{N} \end{array}\right)+\left(\begin{array}{c} N_{D}\left(t\right)\\ N_{S}\left(t\right) \end{array}\right)$$

**Figure 3:**
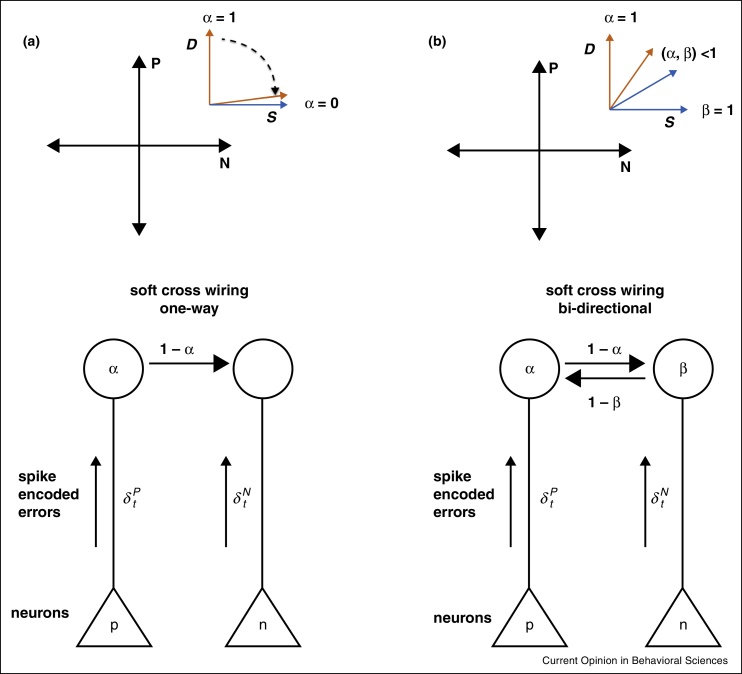
Positive and negative valence prediction systems with soft cross-wiring. Valence predicting systems *P* and *N* help build representations of reward predicting value (*P*) and aversive predicting value (*N*) according to standard reinforcement learning framework (Equations 1–8). There is substantial evidence that midbrain dopaminergic neurons emit reward prediction errors $\delta_{t}^{P}$ in this manner and there is scattered evidence that there is a near-opponent system (*N*) that approximately anti-correlates with the dopaminergic system and is thus capable of the same kind of prediction but for future aversive stimuli. Such predictions are ideally suited to inform an agent when to avoid stimuli or wait, and have been hypothesized to be one substrate for conditioned inhibition. Serotonin (*S*) is thought to be one such opponent system to dopamine. The soft cross-wiring claim is that these systems interact at the level of their neurotransmitter — by virtue of importing both transmitters at their terminals, each system influences the extracellular dynamics of both transmitters. Soft-cross wiring can be thought of as a rotation in the abstraction depicted here for *P* and *N*. A brief thought experiment helps. Imagine that for the *P* and *N* pathways, only *P* contained neurotransmitter (*D*, orange). Both pathways can still encode prediction errors in changes in spike rate but only the *P* pathway translates this modulation into a change in neurotransmitter release (dopamine) while the *N* pathway spikes run out into neurotransmitter-free terminals. Now start moving the dopamine one molecule at a time from the *P* terminal into the *N* terminal and continue until all the dopamine has been moved. At this point the dopamine will now fluctuate as a function of the aversive prediction errors produced in the *N* pathway. At the start of this transfer, dopamine fluctuated according to the reward prediction errors because it was all in the *P* terminals. In the abstract valence space where *P* and *N* point in different directions, this procedure rotates the signal carried by dopamine from the direction of *P* to the direction of *N*.

*α* is the fraction of the native neurotransmitter (labeled *D* here for ‘dopamine’) in the *P* pathway that is present in the *P* terminal and the remainder (1 − *α*) is assigned to the *N* pathway terminal. Similarly, *β* is the fraction of native neurotransmitter (labeled *S* for ‘serotonin’) in the *N* pathway terminal and the remainder (1 − *β*) is assigned to the *P* pathway terminal. Noise terms *N*_*D*_(*t*) and *N*_*S*_(*t*) for each transmitter include synaptic noise and unaccounted for extrasynaptic sources/sinks for *D* and *S*.

Equation 9 expresses two couplings: (1) the coupling of spike-encoded prediction errors along *P* and *N* pathways to perturbations in neurotransmitter release, and (2) the neurotransmitter coupling between the two systems; a feature we have termed soft cross-wiring. For *α* = *β* = 1, the ‘normal’ situation ensues where dopamine and serotonin separately carry the reward prediction error and aversive prediction error information. Ignoring issues about diffusion, this is the situation where the prediction errors from moment-to-moment could be monitored separately by recording dopamine and serotonin simultaneously in the vicinity (receptors could do this). Once cross-loading occurs because either or both alpha and beta deviate from 1, then the prediction errors and the learned weights that instantiate them become mixed. One can then imagine wanting to unmix these signals or detect both serotonin and dopamine in a combination that had computational relevance. We explore this below.

One way to understand the nearly anti-correlated responses of the *P* and *N* pathways is to imagine that these systems’ sensitivities are close in order to discriminate valence in a world where the positive and negative valuations pertinent for survival are close. So let us take the closeness as evidence of an adaptation to a tough set of valence discrimination problems — ignoring the fact that a nervous system does not simply discriminate raw valence but instead assigns it to objects (including living objects), which possess a range of other properties not considered here. Consequently, the near redundancy along the *P* and *N* pathways is not the best representation to process the valence information since it wastes resources because of the high degree of correlation between the systems. One way to deal with this correlation is to decorrelate the signals. One simple way to accomplish this is to rotate to a different set of directions with the obvious ones being *P* + *N* and *P* − *N*. Downstream receptors can easily effect such a transformation either by direct binding or through convergence onto intracellular signaling cascades. This new {*P* + *N*, *P* − *N*} basis provides natural directions where *P* + *N* is a *salience signal* and *P* − *N* a *valence contrast signal*. It is important to note that both dopamine and serotonin would be involved in coding the response along each new direction. Taking the system in Equation 9 expressed in the {*P*,*N*} basis, we can rotate into the {*P* + *N*, *P* − *N*} basis but keeping things expressed in terms of the changes in transmitters to make clear how downstream effector mechanisms could ‘sense’ valence responses along directions that incorporated an efficient encoding principle [9, 10••, 11•, 12, 13]:

(10) $$\frac{1}{\sqrt{2}}\left(\begin{array}{cc} 1 & -1\\ 1 & 1 \end{array}\right)\left(\begin{array}{c} \delta D\left(t\right)\\ \delta S\left(t\right) \end{array}\right)=\frac{1}{\sqrt{2}}\left(\begin{array}{cc} 1 & -1\\ 1 & 1 \end{array}\right)\left\{\left(\begin{array}{cc} \alpha & 1-\beta \\ 1-\alpha & \beta \end{array}\right)\left(\begin{array}{c} \delta_{t}^{r}\\ \delta_{t}^{a} \end{array}\right)+\left(\begin{array}{c} N_{D}\left(t\right)\\ N_{S}\left(t\right) \end{array}\right)\right\}$$

Multiplying out the left hand side shows in Equation 11 how downstream receptors could sense and respond to dopamine and serotonin changes in a manner aligned with *P* − *N* and *P* + *N* directions. They can simply respond to the sum or difference in the fluctuations. This could take place on the surface of a cell or using intracellular cascades (both serotonin and dopamine couple to g-protein coupled receptors for example):

(11) $$\frac{1}{\sqrt{2}}\left(\begin{array}{c} \delta D\left(t\right)-\delta S\left(t\right)\\ \delta D\left(t\right)+\delta S\left(t\right) \end{array}\right)=\frac{1}{\sqrt{2}}\left(\begin{array}{cc} 1 & -1\\ 1 & 1 \end{array}\right)\left\{\left(\begin{array}{cc} \alpha & 1-\beta \\ 1-\alpha & \beta \end{array}\right)\left(\begin{array}{c} \delta_{t}^{r}\\ \delta_{t}^{a} \end{array}\right)+\left(\begin{array}{c} N_{D}\left(t\right)\\ N_{S}\left(t\right) \end{array}\right)\right\}$$

We can express the situation, ignoring lots of potential complications, as:

(12) $$\left(\begin{array}{c} valence\;diff\\ salience \end{array}\right)∼\underset{\begin{array}{c} receptors\;can\;sense\\ sum\;and\;difference\\ of\;5H\;,DA \end{array}}{\underset{︸}{\left(\begin{array}{c} \delta D\left(t\right)-\delta S\left(t\right)\\ \delta D\left(t\right)+\delta S\left(t\right) \end{array}\right)}}∼(Rotate)(Mix)\underset{\begin{array}{c} spike\\ encoded\\ errors \end{array}}{\underset{︸}{\left(\begin{array}{c} \delta_{t}^{r}\\ \delta_{t}^{a} \end{array}\right)}}$$

Notice that for the normal case of *α* = *β* = 1, there is no mixing and the transformation here is a simple decorrelation that would define a salience channel and a valence difference channel. We have left off the issue of how to adjust the sensitivity for the different directions in order to focus on the idea of handling the valence processing problem in terms of efficient encoding and separate value systems (but see [10••, 11•, 13]). Once the mixing matrix above moves away from the identity, then the system mixes the information channels before releasing transmitter and before decoding by downstream receptors. We strongly suspect that this coupling has many consequences only a few of which are sketched here, but it is possible that such mixing allows the system to learn to predict composite values of events that predict both future rewards and punishments. The current idea and its scant but supporting data suggest that both dopamine and serotonin play an important information-bearing role in learning such composite values.

There is a loose, but instructive analogy here with the ecology of (color) vision and red/green cone sensitivities. In primates, the peak spectral sensitivities of red and green cones are very close (∼30 nm), which apparently reflect the range of wavelengths where such discrimation is computationally pertinent [10••, 11•]. The proximity of the spectral sensitivity peaks for *R* and *G* pathways is put into perspective when the response properties of these pathways is faced with measured visual statistics from the natural world of the primate [10^••^]. This work in vision has relied in part on the capacity to capture images of natural visual scenes easily and cheaply; however, collecting natural reward statistics is subtle and ultimately involves the fluctuating internal needs of the mobile creature as they compare to the surrounding environment.

## Summary

In summary, we began with a new measurement of striatal dopamine in human subjects and found that existing computational models of dopaminergic function were inadequate to capture the possibility that dopamine encodes prediction errors in reward and counterfactual errors in reward. One trivial possibility is that some simplistic element of the behavioral task (a scalar betting game against a market) is accidentally creating a situation for dopamine release that is not normal and only appears to encode a bet-dependent counterfactual signal — this is indeed possible since the subjects involved have a disease of their dopaminergic system (Parkinson's Disease). The interactions that we posit here are quite specific in terms of transmitters and neural elements; however, other work has observed loosely similar coupling and suggested a way to relate l-DOPA drugs used to treat Parkinson's and computational ideas about basal ganglia function [22, 23, 24, 25]. We suspect that there are ways to connect the framework sketched here to the ideas present in that work.

Several new possibilities emerge from the opinion presented here. First, we suggest that all stimuli have the possibility to be assigned a composite of positive and negative valence through the operation of two prediction systems *P* and *N* (positive and negative) dedicated to making this assignment. We think here of *P* and *N* as directions in some valence space and claim that they rate the reward-predicting or aversive-predicting valence of situations that represent difficult valence detection problems. These same systems can share transmitter at their terminals when those terminals happen to be sufficiently close; a fact that immediately mixes reward and aversive prediction information. In the context of the behavioral task in Figure 1, this sharing provides the explanation for the observed counterfactual component. As outlined above, we see that soft cross wiring might also allow the system to rotate parametrically from the {P,N} to other bases; we considered one specific case. One possibility is that the bet dependence of dopamine encoding of prediction errors is directly related to the coupling coefficients in Equation 9.

We have completely avoided treating learned timing among stimuli and the near-term rewards and punishments that they portend. However, there should be very interesting connections of this framework to related analyses in the visual system. For example, there is strong evidence that independent objects in the visual world are the source of natural visual scaling statistics [26^•^] and that visual cortical neurons can learn to respond to reward-predictive visual cues [27^•^]. We suspect that an analysis of this coupling based on an efficient encoding framework [11^•^] would show that many levels of structure in visual pathways should show predictable *P* and *N* channel modulation. It could even be the case that the exquisite structural arrangements in the striatum can be understood as natural ways to organize *P* and *N* information in a fashion homologous to similar analyses in visual cortex [11•, 13].

## Conflict of interest statement

Nothing declared.

## References and recommended reading

Papers of particular interest, published within the period of review, have been highlighted as:

• of special interest
•• of outstanding interest
